# Pediatric Mechanical Circulatory Support: Pathophysiology of Pediatric Hemostasis and Available Options

**DOI:** 10.3389/fcvm.2021.671241

**Published:** 2021-09-01

**Authors:** Chiara Giorni, Alessandra Rizza, Isabella Favia, Antonio Amodeo, Fabrizio Chiusolo, Sergio G. Picardo, Matteo Luciani, Giovina Di Felice, Luca Di Chiara

**Affiliations:** ^1^Pediatric Cardiac Intensive Care Unit, Department of Cardiology and Cardiac Surgery, Bambino Gesù Children's Hospital, Istituto di Ricovero e Cura a Carattere Scientifico, Rome, Italy; ^2^Mechanical Circulatory Support Unit, Department of Cardiology and Cardiac Surgery, Bambino Gesù Children's Hospital, Istituto di Ricovero e Cura a Carattere Scientifico, Rome, Italy; ^3^Department of Anesthesia and Critical Care, Anestesia Rianimazione Comparto Operatorio, Istituto di Ricovero e Cura a Carattere Scientifico, Rome, Italy; ^4^Department of Oncohematology, Haemostasis and Thrombosis Center, Bambino Gesù Children's Hospital, Istituto di Ricovero e Cura a Carattere Scientifico, Rome, Italy; ^5^Hemostasis Laboratory, Bambino Gesù Children's Hospital, Istituto di Ricovero e Cura a Carattere Scientifico, Rome, Italy

**Keywords:** mechanical circulatory support, anticoagulation, pediatrics, bleeding, thrombosis, heparin, bivalirudin, quality improvement

## Abstract

Pediatric mechanical circulatory support (MCS) is considered a strategy for heart failure management as a bridge to recovery and transplantation or as a destination therapy. The final outcome is significantly impacted by the number of complications that may occur during MCS. Children on ventricular assist devices (VADs) and extracorporeal membrane oxygenation (ECMO) are at high risk for bleeding and thrombotic complications that are managed through anticoagulation. The first detailed guideline in pediatric VADs (Edmonton Anticoagulation and Platelet Inhibition Protocol) was based on conventional antithrombotic drugs, such as unfractionated heparin (UFH) and warfarin. UFH is the first-line anticoagulant in pediatric MCS, although its profile is not considered optimal in pediatric setting. The broad variation in heparin doses among children is associated with frequent occurrence of cerebrovascular accidents, bleeding, and thrombocytopenia. Direct thrombin inhibitors (DTIs) have been utilized as alternative strategies to heparin. Since 2018, bivalirudin has become the chosen anticoagulant in the long-term therapy of patients undergoing MCS implantation, according to the most recent protocols shared in North America. This article provides a review of the non-traditional anticoagulation strategies utilized in pediatric MCS, focusing on pharmacodynamics, indications, doses, and monitoring aspects of bivalirudin. Moreover, it exposes the efforts and the collaborations among different specialized centers, which are committed to an ongoing learning in order to minimize major complications in this special pediatric population. Further prospective trials regarding DTIs in a pediatric MCS setting are necessary and in specific well-designed randomized control trials between UFH and bivalirudin. To conclude, based on the reported literature, the clinical use of the bivalirudin in pediatric MCS seems to be a value added in controlling and maybe reducing thromboembolic complications. Further research is necessary to confirm all the results provided by this literature review.

## Introduction

Patient population on end-stage heart failure has grown significantly during the last two decades. Independently from the different etiologies of heart failure, unresponsiveness to conventional medical therapy must be an indication for considering mechanical circulatory support (MCS) as a strategy to improve end-organ perfusion, which has resulted in better quality of life and survival for both patients on destination therapy and those bridged to transplant. Additionally, evidence for heart recovery has been even more reported for both adult and pediatric populations. Modulating activation of the coagulation cascade due to exposition to extracorporeal circuit is of basic relevance for a successful strategy of mechanical assistance where hemorrhagic and thrombotic events remain significant complications that negatively influence morbidity and mortality. Peculiarities of coagulation in neonates and congenital heart disease (CHD) patients make handling of coagulation particularly challenging and even more frequently requiring clinicians experienced in hemostasis in this field. The large number of heart centers dealing with MCS results in a fragmentation of clinical experience, which requires an effort for close collaboration in sharing data and protocols for coagulation management on MCS. This article provides a short description of different types of MCS: their differences in terms of nomenclature, indications, short- and long-term strategies, positioning and modality of implantation, technical characteristics, and type of blood flow generated. Particular attention is focused on describing pharmacological and monitoring issues related to both traditional and alternative antithrombotic therapy. Efforts in collaboration between different specialized centers committed to an ongoing learning to minimize major complications in this special pediatric population are outlined. The original contribution of this article includes our lifelong experience in managing anticoagulant therapy for different types of MCS in a setting of heterogeneous patient population for age, weight, and diagnosis. On the basis of the most recent internationally shared protocols, we try to give additional recommendations regarding the antithrombotic therapy for ventricular assistance devices.

## General Features and Nomenclature of MCS Systems

MCS is a generic acronym that currently describes mechanical devices able to pump blood (1). Specific definitions for different MCS types are applied, depending on the purpose, the duration, the cannulation, and the type of flow.

Extracorporeal membrane oxygenation (ECMO) is generally indicated as a short-term bridge to recovery or to organ transplantation for patients affected by cardiac or pulmonary failure refractory to medical therapy. ECMO can provide respiratory support or combined respiratory and cardiac support.

Veno-arterial (VA) ECMO is based on an extracorporeal circuit with a (roller or centrifugal) pump draining blood from the systemic venous pole of circulation (right atrium, superior and/or inferior vena cavae), flowing it through a gas exchange (oxygenator), and rewarming and returning it to central systemic arteries (aorta, carotid, and/or femoral arteries). This blood flow through the ECMO circuit results in a more or less effective systemic venous decongestion with an increase in cardiac output. The presence of an oxygenator allows for optimization of hemoglobin oxygenation and replacement of lung function in terms of gas exchange. The final effect on systemic oxygen delivery is the result from residual native cardiac output integrated with required pump flow. The final coronary oxygen delivery will depend on anterograde blood ejected by the left ventricle (whose oxygenation will be influenced by the gas exchange capability of lungs) and the quota of fully oxygenated pump flowing back in the ascending aorta from arterial cannula.

According to the site of vessel cannulation, ECMO support may be classified as central (direct right atrial and aortic cannulation through central sternotomy) and peripheral (by cannulating veins and arteries from the neck and/or groin).

This mechanically driven bypass of the pulmonary circulation will result in left ventricle afterload increase, which could precipitate left ventricle systolic performance and pulmonary edema.

Veno-venous ECMO is based on blood draining from and returning into the venous system. The final effect is an augmentation of central venous saturation on the basis of pump flow and oxygenator gas exchange. VV ECMO can provide only an oxygenation function, but not a pump support. Thus, the main prerequisite for establishing the VV ECMO is an adequate cardiac function and the absence of a lung barrage related to high pulmonary vascular resistances.

Regarding ventricular assist devices (VADs), the terminology is evolving and varies upon the type of cardiac assistance. VADs can support left (left VAD) or right (right VAD) ventricle or both (bi-VAD) by directly unloading the failing chamber and by returning blood to either the aorta (left VAD) or pulmonary artery (right VAD). The pump can be placed outside (paracorporeal) or implanted (intra-corporeal) inside the body. Different flow types are generated depending on the technical features (e.g., pulsatile, non-pulsatile, continuous centrifugal, and continuous axial). According to pump flow dependence from pharmacological manipulation of systemic vascular resistances, left VADs with continuous flow may result in a more complete left ventricle unloading compared to ECMO and pulsatile VAD. Moreover, VADs can be categorized into those which allow for patient discharge (durable devices) and those requiring patient hospitalization (1, 2). Lastly, short-term devices are those utilized for organ recovery or for bridge to transplantation, whereas long-term devices are those utilized for patients ineligible for transplant as destination therapy (3).

**Figure 1:**
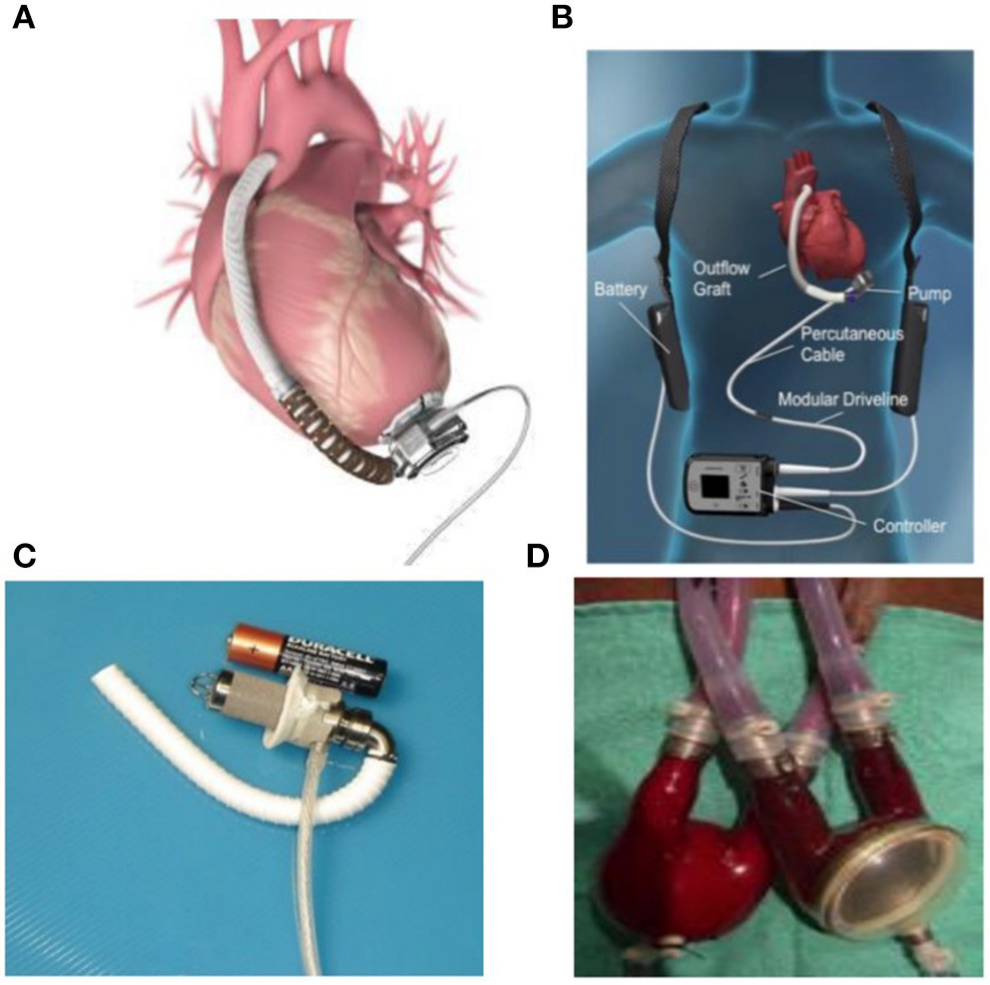
Durable devices: **(A)** Heartware **(B)** Heartmate3 **(C)** Jarvik infant **(D)** Berlin Heart.

The main characteristics of available MCS, depicted for their main technical features and clinical indications, are described in Table 1 and durable devices are presented in Figure 1.

**Table 1:** Main characteristics of available MCS devices.

**Indication**	**Name**	**Placement**	**Pump mechanism**	**Type of flow**	**Max flow**	**Support type**	**Used in children**	**Description of mechanics**
Short-term devices	VA-ECMO	PercutaneousSternotomy	CentrifugalRoller	Continuous	10 L/min	Biventricular, temporary	Yes>2–2.5 kg	Impeller with magnetically suspended pump components, with minimal direct contact and friction. Oxygenator.
	Centrimag^TM^/ Pedimag^TM^	Paracorporeal	Centrifugal	Continuous	5 L/min	Left, right or biventricular, temporary	YesPedimag^TM^: 10–15 kgCentrimag^TM^: >15 kg	Combined design with drive, magnetic bearing, pump magnetically levitated rotor with no valves, seals and mechanical bearings.
	ProTek DUO®Cannula size 21Fr	Percutaneous	Centrifugal	Continuous	4.5 L/min	Right, temporary	Adolescents>40 kg	Continuous centrifugal flow via dual lumen cannula featuring right atrium to pulmonary artery bypass with insertion at the right jugular vein
	Impella® 2.5/CP/5.0Cannula size13 Fr-14 Fr-22 Fr	Percutaneous	Axial flow	Continuous	2.5–5 L/min	Left or right, temporary	YesType of Impella depends on arterial's patient size	Arterial percutaneous cannulation, microaxial minimally invasive pump based on the principle of Archimedes screw, which directly unloads the left or right ventricle, increasing coronary flow
Long-term devices	Syncardia TAH	Sternotomy	Pneumatic	Pulsatile	7–9 L/min	Biventricular, durable	Adolescents	Pulsatile system totally implantable that surgically replaces the heart. Two pneumatically driven ventricles connected to external pump through percutaneous lines.
	Berlin Heart EXCOR®	Sternotomy	Pneumatic	Pulsatile	3–7.2 L/min	Left, right orBiventricular, durable	Yes>3 kg	Pneumatically driven pulsatile pump (divided into two blood and air chambers) with a driving unit.
	Jarvik 2000 Infant Jarvik 2015	Sternotomy	Axial	Continuous	7 L/min3 L/min	Left, durable	Adolescents >30 kg>8 kg	Blood pump, axial rotor supported by ceramic bearings, Dacron-outflow graft, percutaneous power cable, pump-speed controller, and direct-current power supply.
	HeartMate II^TM^	Sternotomy	Axial	Continuous	3–10 L/min	Left, durable	Adolescents	Continuous axial flow driven by rotor, ball and cup bearings.
	HeartMate 3^TM^	Sternotomy	Centrifugal	Continuous	10 L/min	Left, right, or biventricular, durable	Adolescents>30 kg	Fully magnetically levitated rotor with wide blood-flow gaps; contactless and frictionless rotor minimizes shear stress
	HeartWare	Sternotomy	Centrifugal	Continuous	10 L/min	Left, durable	Yes>15 kg	Small miniature centrifugal wearless pump, and hydro-magnetic suspension rotor, allowed for pericardial placement

*MCS, mechanical circulatory support; VA, veno arterial; ECMO, extracorporeal membrane oxygenation; TAH, total artificial heart*.

## Effects of the Blood-Surface Interface on Coagulation

Endothelial cells produce anticoagulant and procoagulant factors that act together in order to maintain homeostasis (Table 2) (4). Blood exposure to non-endothelialized biomaterials of extracorporeal artificial tubing activates a defense reaction that may vary in intensity from patient to patient. This reaction results in significant modifications of the physiologic coagulation balance by consuming anticoagulation factors and stimulating production of procoagulant ones. Additionally, activation of the complement and contact pathway induces production of inflammatory cytokines.

**Table 2:** Endothelial cell properties.

**Anti-coagulant factors produced/released by ECs**
- NO, prostacyclin
- heparin sulfates
- thrombomodulin
- t-PA
- urokinase plasmin activator
**Anti-coagulant actions promoted by ECs**
- synthesize TFPI
- show negative charge that repel platelets
- produce PC receptor
- bind annexin V, which binds phospholipids
**Pro-coagulant factors produced/released by ECs**
- endothelins
- adhesive proteins: vWF, collagen, fibronectin, vitronectin, laminin
- TF
- PAI-1
**Pro-coagulant factors expressed by systemic ECs**
- P and E selectins, PECAM
- ICAM and VLA counter-receptors
**Pro-coagulant factors expressed by pulmonary ECs**
- produce TxA2
- express receptors for factor V, IXa, and Xa and fibrinogen
- bind fibrinogen through integrins
- reduce surface thrombomodulin
- contain intracellular adhesion molecules

*NO, nitric oxide; t-PA, tissue plasminogen activator; ECs, endothelial cells; vWF, von Willebrand factor; TFPI, tissue factor pathway inhibitor; PC, protein C; TF, tissue factor; PAI, plasminogen activator inhibitor; PECAM, platelet endothelial cells adhesion molecule; ICAM, intercellular adhesion molecule; VLA, very late antigen; TXA2, thromboxane A2*.

Activation of the contact pathway of coagulation provides potential new targets for anticoagulant therapies (5).

## The Challenges of Pediatric MCS: Coagulative Complications

Compared to adult population, pediatric MCS is negatively characterized by higher incidence of hemorrhagic and thrombotic events, and this may be explicated in terms of hemostasis alterations related to developmental age and the presence of CHD.

Retrospective data from ELSO registry report a 39% prevalence of hemorrhagic complication in the pediatric population (6).

Major bleeding complications and strokes have been, respectively, reported with an incidence until 50 and 29% in the Berlin Heart (BH) Excor® pediatric population (7). Similar to pulsatile flow devices, such as Berlin Heart EXCOR, in patients implanted with paracorporeal continuous-flow devices, such as the Rotaflow (Maquet, Wayne, NJ) and the Centrimag/Pedimag (Abbott, Abbott Park, IL), the rate of CVE remains up to 24% (8).

Regarding continuous-flow durable devices implanted in adolescents, the adverse event rate, especially for neurologic dysfunction, including strokes, was registered relatively low at 10%, with most of the events occurring in the first 3 months after implantation (9).

## The Challenges of Pediatric Coagulation: The Developmental Hemostasis and CHD-Related Modifications in Hemostasis

The developmental hemostasis increases the risk for hemorrhagic complications in neonates and infants and offers additional challenges in titration of standard anticoagulation in pediatrics requiring MCS.

The term “developmental hemostasis,” introduced by Maureen Andrew in 1980, describes the physiological changes of the coagulation system related to age (10), where functional levels of coagulation proteins “change in a predictable way with age” (11).

Neonates have physiologically low levels of vitamin K-dependent and contact factors (FXI, FXII, prekallikrein, and high-molecular-weight kininogen) that gradually increase to values approaching adult levels by 6 months of life (10, 12). Physiological levels of plasmatic fibrinogen, FV, FVIII, and FXIII have been reported while levels of von Willebrand factor (vWF) and FVIII are increased at birth and for the first 3 months of life (12). Decreased anticoagulant factors including protein C and S, and antithrombin (AT) as well as decreased thrombin generation and reduced clot lysis have been described in neonates.

The reduced levels of coagulation factor in infants results in alteration of conventional tests such as the activated partial thromboplastin time (aPTT). Unfortunately, there is no standardization for aPTT because of a wide variability of reagents and analyzing systems from center to center implying different reference ranges (13). Other global tests may have a different level of sensitivity to detect abnormalities in hemostatic proteins related to age. For example, the thromboelastography (TEG) parameters in normal children do not differ compared to those in adults (14).

Moreover, acquired coagulation defects have been reported in children with cardiac disease. In children with CHD, a 48-month delay in normalization of coagulation factors has also been documented. Children with cyanotic defects are much more likely to be polycythemic and manifest a hypercoagulability when younger (with increased platelets, fibrinogen, FV, and FVIII) (15).

In cyanotic older children, chronic hepatic congestion or heart failure may lead to liver dysfunction and a decreased production of coagulation factors and platelets in addition to an increased fibrinolytic activity (15).

## The Challenges of Pediatric MCS: Hemocompatibility and Pump Designs

Efforts in reducing hemorrhagic and thrombotic events in pediatric MCS population have involved optimization of hemocompatibility of artificial surfaces and pumps, and pharmacologic manipulation of coagulation.

Biomaterials or artificial substance of MCS in contact with human body are engineered in order to optimize biocompatibility. Biocompatibility refers to the effects that a biomaterial has on host tissues that are exposed to it. Hemocompatibility, a subset of biocompatibility, describes the effects of a MCS on the coagulation and inflammatory system. The ideal biocompatible surface for blood is the uninjured endothelium. Nowadays, different biomaterials are available for ECMO circuits, and others are still in the process of research and development, in order to target thrombin inhibition and to inhibit platelet adhesion/activation. These biomaterials have been recently classified into three categories: biomimetic surfaces [heparin, nitric oxide (NO), and direct thrombin inhibitors (DTIs)]; bio-passive surfaces [phosphorylcholine (PPC), albumin, and poly-2-methoxyethylacrylate (PMEA)], and endothelialization of blood-contacting surfaces (16).

A contribution to clotting activation also derives from *hemolysis* caused by the pumps' direct physical injury and the shear stress forces on red blood cells. Hemolysis (4) is characterized by plasma-free hemoglobin (PfH) > 40 mg/dl (1) and increased lactate dehydrogenase (LDH) levels. *Shear stress* is a known cause leading to von Willebrand factor (vWF) emergence. vWF is a multimeric glycoprotein containing binding sites for both platelet glycoprotein receptors and collagen (17). The adhesive activity of vWF depends on the size of its multimers. The high-molecular-weight multimers (HMWMs) interact with collagen and platelet receptors under the condition of shear stress (17). Interaction of PfH with the vWF plays a significant role in VAD-related thrombosis (1). Close PfH and LDH monitoring can be helpful in anticipating this adverse event (1). Instead, cleavage and consumption of the HMWM bound to platelets and collagen can trigger acquired von Willebrand syndrome (AVWS) with potential risk for bleeding. There are few available studies suggesting that AVWS is a common and underdiagnosed clinical feature in pediatric MCS (17–20). Optimization of VAD pump designs has been proven to improve hemocompatibility and lower adverse event occurrence. Roller pumps and both short- and long-term axial flow devices induce higher level of hemolysis compared to intracorporeal or extracorporeal centrifugal flow devices. In literature, higher baseline levels of hemolysis have been reported with the HeartMate II compared to the Heartware, and patients treated with Heartmate III have shown less HMWM loss compared with HeartMate II (21). According to these reported data, it seems to be quite evident that the choice for a certain device might affect the risk for major clinical events. Nevertheless, in pediatrics, the choice for a VAD is sometimes a forced choice. Indeed, the type of device implanted depends on the patient's weight: pulsatile flow devices are implanted in patients with a weight of < 15 kg, while continuous-flow devices are not available for younger children, but only for those with a weight of more than 15–17 kg (22). Since the recipients of the pulsatile flow devices are more frequently affected by cardiac failure in CHD, this is the main indication for pulsatile flow devices in a pediatric population.

## The Challenges of Pediatric MCS: Anticoagulants

### Unfractionated Heparin

Nowadays, the major complications persist and continue to potentially lead to morbidity and mortality, in spite of the extensive use and research in MCS (23). Anticoagulant strategies associated or not with anti-platelet agents have a central role in balancing coagulation and preventing major hemorrhagic or thrombotic events.

Conventional anti-thrombotic regimens involve unfractionated heparin (UFH), low-molecular weight heparin (LMWH), or warfarin with the addition of anti-platelet agents (acetylsalicylic acid and clopidogrel). However, it has to be remarked that no standardized guidelines for pediatric patients are currently available. DTIs such as bivalirudin are becoming a common alternative to conventional anticoagulation in pediatric patients.

The anticoagulation strategy of choice and its titration and monitoring constitute additional challenges due to the peculiar pharmacokinetics and pharmacodynamics in children, with supplementary difficulty in neonates.

### Structure and Mechanism of Action

**Figure 2:**
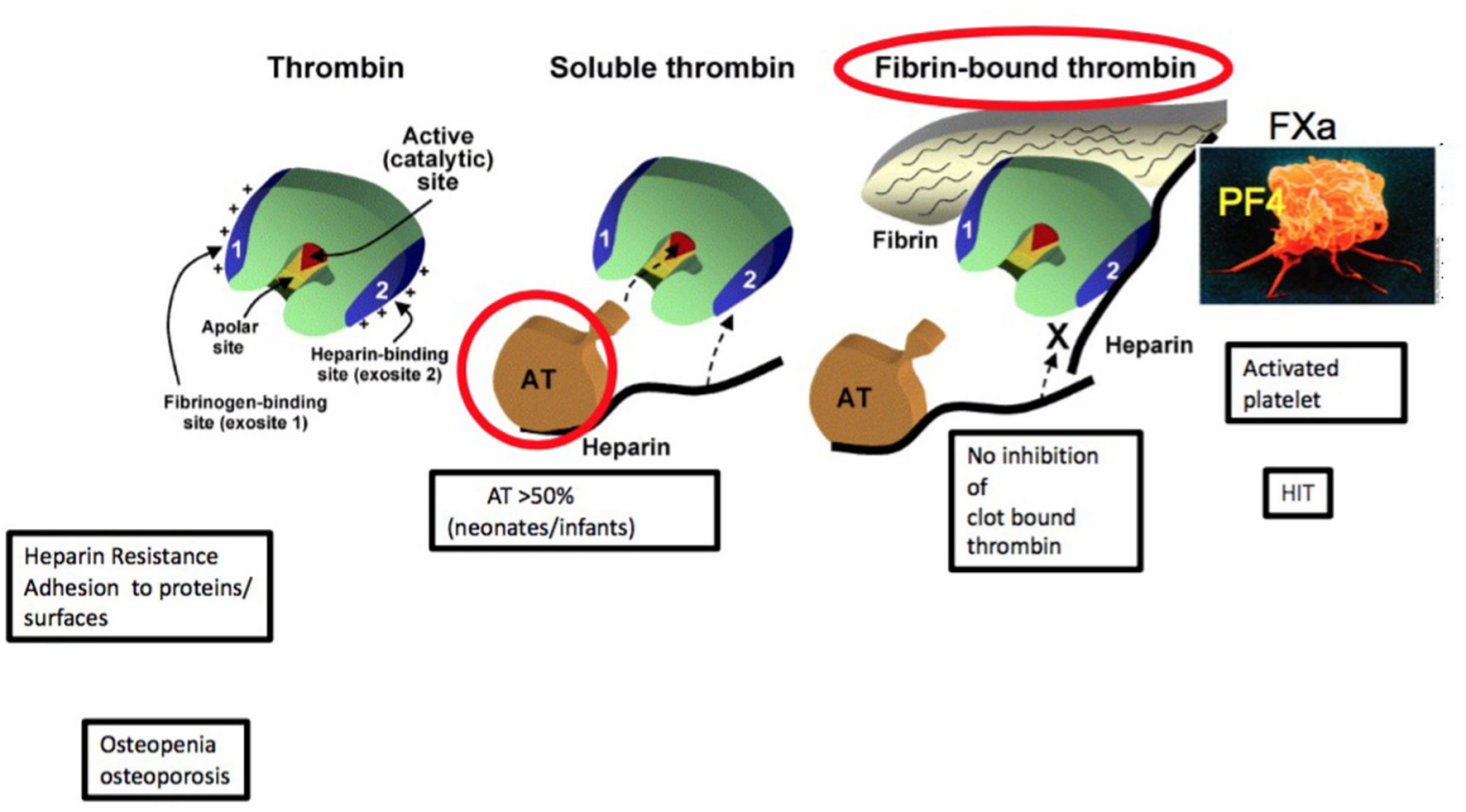
Heparin mechanism (Modified by Patricia Massicotte. Warkentin TE. Best Practice & Research Clinical Haematology, 2004).

Heparin is a sulfated mucopolysaccharide derived from porcine intestine. It is a heterogeneous molecule regarding size, anticoagulant activity, and pharmacokinetic properties. Heparin molecules range in molecular weight from 3,000 to 30,000 kDa. Just one-third of the heparin molecules has the unique pentasaccharide sequence, which is responsible for the anticoagulant effect of heparin. The heparin/antithrombin (AT) complex inactivates thrombin, factor IIa, and factors Xa, IXa, XIa, and XIIa. Heparin catalyzes AT-mediated thrombin inhibition by a ternary heparin/AT/thrombin complex. Instead, in order to inhibit the factor Xa, it is necessary to bind only AT. In both cases, the unique pentasaccharide sequence, found within some heparin molecules, is needed. Heparin prevents fibrin formation and inhibits thrombin-induced activation of platelets and factors V, VIII, and XI, by inactivating or decreasing thrombin generation (24). UFH inhibits thrombin only after it is generated, but it is not a barrier for the thrombin generation in case it is already bound to fibrin (Figure 2). In animals, it has been shown that the link of heparin with platelets and endothelial cells may trigger heparin-induced bleeding, by altering capillary integrity and permeability to plasma proteins and eventually causing microvascular bleeding (25).

### Limitations of Heparin

Heparin has various significant limitations, particularly evident in infants.

1. First, heparin has a high degree of inter- and intra-patient variability in dosing, which leads to poor maintenance of standard therapeutic levels (26). This is a particular concern for infants due to the highly variable AT levels (27).
2. Second, monitoring heparin with the aPTT or anti-factor Xa are problematic, due to the limitations of these assays. The aPTT, in particular, is frequently impacted by these variables: pre-analytic (blood sampling), analytic variables (reagent and/or coagulometer used), and biological variables (lupus anticoagulant, fibrinogen concentration, factor XII deficiency that falsely prolongs aPTT, and AT deficiency or increased levels of acute-phase reactants that falsely decrease aPTT) (28).
3. Third, heparin can induce thrombocytopenia. However, the incidence of heparin-induced thrombocytopenia (HIT) in neonates, particularly those with CHD, is about 1%, which is not insignificant considering that this complication can lead to devastating consequences including death (29).
4. Fourth, heparin is a biologic compound; it is subject to potential contamination (30).
5. Finally, heparin inhibits osteoblast formation promoting bone loss and osteoporosis (31).

### Heparin Monitoring

Problems related to balance coagulation on MCS are associated to the accuracy in monitoring heparin administration.

Different monitoring tests are used to measure heparin effect.

1. The activated clotting time (ACT) is a very basic test that measures clotting time in seconds in whole blood, by using one of two activators (kaolin or celite) (32). ACT is a bedside, rapid, and widely used test to monitor heparin titration on ECMO. Nevertheless, ACT does not solely or accurately reflect the effect of heparin. Multiple factors can prolong the ACT independently of UFH dosing, including hemodilution, platelet dysfunction or count, hypothermia, hypofibrinogenemia, and coagulation factor deficiencies (33).
  The safety and efficacy of ACT to monitor heparin anticoagulation in children have not been really evaluated by well-designed trials.
2. The aPTT is a standard global clotting assay that reflects the function of contact factors (FXI, FXII) and FII, FVIII, FX, up to the conversion of fibrinogen to fibrin. aPTT evaluates the intrinsic and common pathways of the coagulation cascade. It is performed by adding an activator and phospholipid to citrated platelet-poor plasma and then adding calcium to measure clotting time in seconds (33). This test is usually used to evaluate the effect of different anticoagulant drugs, such as DTIs and UFH (34). Monitoring of UFH has been based on aPTT (24, 32).
3. The anti-factor Xa assay is a test to monitor heparin effect that has recently gained popularity. Patient plasma is added to a test reagent that contains excess of factor Xa. UFH from the patient's plasma binds AT and inhibits Xa. In this way, it measures the residual amount of Xa linked to a chromophore substrate, which will be inversely proportional to the UFH concentration. The amount of residual Xa is used to calculate the anti Xa level (28).

Regarding heparin monitoring in pediatric ECMO, several limitations of each test have been evidenced as well as a poor correlation among them. ACT is a bedside, rapid, and widely used test to monitor heparin titration on ECMO. Currently, 94% of ELSO-reporting centers check aPTT at least once per day (34). Nevertheless, arguments against the use of aPTT alone in accurate assessment of heparin dose regimen have been reported. Evidence-based guidelines for UFH monitoring in adults recommend a therapeutic aPTT range, which is determined by its correlations with therapeutic UFH levels and measured by anti Xa assay (range 0.35–0.7 UI/ml) or protamine titration (range 0.2–0.4 UI/ml) (28). However, in children, a protamine titration range of 0.2–0.4 UI/ml appeared equivalent to a wider anti-Xa range of 0.17–0.85 UI/ml. Consequently, in pediatric patients, the therapeutic range of aPTT based on anti-Xa assay or protamine titration is significantly wider than adult population (28) and consequently less reliable.

In adult ECMO patients, studies relating ACT and aPTT values to heparin concentrations evidenced a poor or moderate correlations between, respectively, ACT, aPTT, and heparin doses. Even a poor correlation between ACT and aPTT values has been reported (35).

In children, Liveris et al. described that 44% of aPTT values and anti-Xa level measurements were discordant (36). Similarly, other studies in pediatric patients on ECMO evidenced a poor correlation between heparin levels and ACT or aPTT values. Hypothetical therapeutic levels of ACT (180–220 s) have been associated to a wide range in anti-Xa (0.3–0.7 IU/ml) (*r* = 0.02) and this was explained with frequent thrombocytopenia and coagulation factor deficiencies characterizing a pediatric patient population. aPTT was also weakly correlated with anti-Xa, although somewhat better than ACT, even in neonatal populations (37). A stronger positive correlation between Anti-Xa levels and heparin dose in case of simultaneous comparison has been described compared to ACT and aPTT (34, 36, 38). Therefore, anti-Xa assay seems to be the more accurate measure of heparin concentration. Nevertheless, anti-Xa levels could be underestimated in the presence of hyperbilirubinemia or high PfH. Levels of PfH of 50 mg/dl significantly decrease the anti-Xa activity (39, 40). The main characteristics of the conventional laboratory tests are shown in Table 3.

**Table 3:** Characteristics of conventional coagulation tests.

**Monitoring tests**	**ACT (s)**	**aPTT (s)**	**Anti Xa (UI/ml)**
Type of test	Measure of clotting time in whole blood Coagulation activated by kaolin or celite Exploration of common and intrinsic pathway Context-sensitive: changes depending on UFH levels but also on temperature, pH, calcium, PLT count, FBN levels, and red blood cell contribution	Platelet-poor plasma Exploration of common and intrinsic pathway Coagulation activated with phospholipids Partially context-sensitive: ignores PLT count but partially depends on FBN concentration	Platelet-poor plasma Measure of the ability of heparin to antagonize F Xa Uses a reagent and a chromophore substrate to quantify the heparin–AT complex Not context-sensitive: ignore PLT count and FBN concentration
Strengths	Results in seconds Bedside test	Rapid results	Results in minutes, not influenced by coagulation factor concentrations
Limitations	Not specific to the effects of heparin	Influenced by deficiencies or defects of intrinsic clotting cascade, vit K deficiency, and Factor VIII	Affected by hyperbilirubinemia and high PfH, hyperlipidemia Highly dependent on serum AT levels

*ACT, activated clotting time; aPTT, activated partial thromboplastin time; anti F Xa, anti Factor X activated; PfH, plasma-free hemoglobin; PLT, platelet; FBN, fibrinogen; UFH, unfractionated heparin; AT, antithrombin*.

### Thromboelastometry/-Graphy (ROTEM^®^/TEG^®^)

Most thrombo-embolic complications in patients receiving a MCS take place in spite of normal values of standard coagulation tests. This is probably related to multiple standard coagulation assay limitations. Traditional coagulation assays are plasmatic tests, which are not able to detect hyper- or hypo fibrinolysis, hypercoagulability due to tissue factor expression on circulating cells, and the platelet functionality and the clot firmness.

Viscoelastic tests, such as thromboelastometry/-graphy, and platelet (PLT) function tests, such as Platelet mapping, Multiplate^®^, and aggregometry, reflect in detail the real hemostatic status of patients, including their specific response to anticoagulation and antiplatelet therapy.

The viscoelastic tests provide a point-of-care assessment of the entire hemostatic pathway in whole blood, from clot initiation to fibrinolysis. A variety of activators are employed for each device to examine different aspects of the coagulation pathway (41). A graphical representation of the parameters is realized as the blood starts clotting, in a similar but not directly interchangeable manner, among the different machines.

Regarding viscoelastic tests, normal values in healthy neonates and older infants have been published (42). In addition, TEG parameters, including functional fibrinogen, have been demonstrated to be comparable between cyanotic and acyanotic children (41).

Therefore, viscoelastic tests and platelet function assays are recommended during MCS to reduce the risk of thrombosis and bleeding (43). Even though many centers have included TEG?/ROTEM? into their anticoagulation algorithm management protocols, there is the necessity of more extensive and well-designed trials in order to specify their role in pediatric MCS.

Even the relevance of PLT function monitoring with aggregometry needs further investigation.

### Heparin Resistance

The progressive increase of heparin dose based on anti-Xa, aPTT, or ACT is defined “heparin resistance.” The mechanism can be dependent on or independent of AT. The mechanism AT mediated includes reduced synthesis, increased clearance consequent to nephropathy, and accelerated consumption [secondary to heparin pretreatment, upregulation of hemostatic system, and mechanical reasons (MCS)]. The AT-independent heparin resistance may be related to increased heparin binding to proteins and platelets (non-specific binding, thrombophilia, PLT activation, or release), medications (such as nitroglycerin), or increased factor VIII (44). Traditionally, heparin resistance management includes the administration of higher doses of heparin, AT concentrate supplementation, or fresh frozen plasma (FFP) transfusion (44).

### Antithrombin Replacement Therapy

Acquired AT deficiency is more common than congenital AT deficiency, which is the only in-label application for AT concentrate administration. The US Pediatric Health Information System provided a review and concluded that 97% of children who were treated with AT received it off-label, particularly in neonates. Congenital heart/lung problems (36%) represented the most common diagnosis associated with off-label use, and the most common setting was ECMO (45). Anyway, data supporting this practice are sparse. The reduction in heparin requirements following AT administration is highly variable and studies of children on ECMO have thus far not shown any beneficial effect of AT administration on clinical outcomes, including bleeding, blood product administration, ECMO circuit changes, length of stay, or mortality (46–48). AT replacement in VAD patients to maintain an AT activity above 70% was recommended by the only guidelines available, which are related to pediatric patients implanted with Berlin Heart Excor and anticoagulated with heparin (49). Usually, AT dosing is computed on patient weight and desired AT activity level [FDA LGBU: ATryn antithrombin (recombinant) prescribing information 1999]. However, the optimal target threshold for AT supplementation in ECMO has not yet been clearly defined. Nelson et al., in a recent international survey, reported a highly variable target AT range, between 30 and 120% (50). Regarding the mode of administration, continuous infusions have been reported to keep AT blood levels more stable compared to bolus dosing in subjects affected by congenital AT deficiency, leading to reduced bleeding complications (51). Yet, there is a lack of data indicating whether the bolus or the continuous infusion of AT impacts clinical outcomes for patients on ECMO. The results of this study indicated that the continuous infusion of AT increased the time that ACT is maintained in therapeutic range, lowered the heparin dose, did not increase hemostatic complications, demonstrated a trend toward fewer heparin dose adjustments, and lowered blood product usage. However, this study had two main limitations: a small sample size (*n* = 14) and a historical case–control design. There was a lack of close examination regarding the relationship between the AT activity and global hemostatic function. This implies the necessity of a research about interactions between AT and other pro- and anticoagulant factors in order to identify clinical indications for the administration of AT.

### DTIs: Differences From Heparins

**Figure 3:**
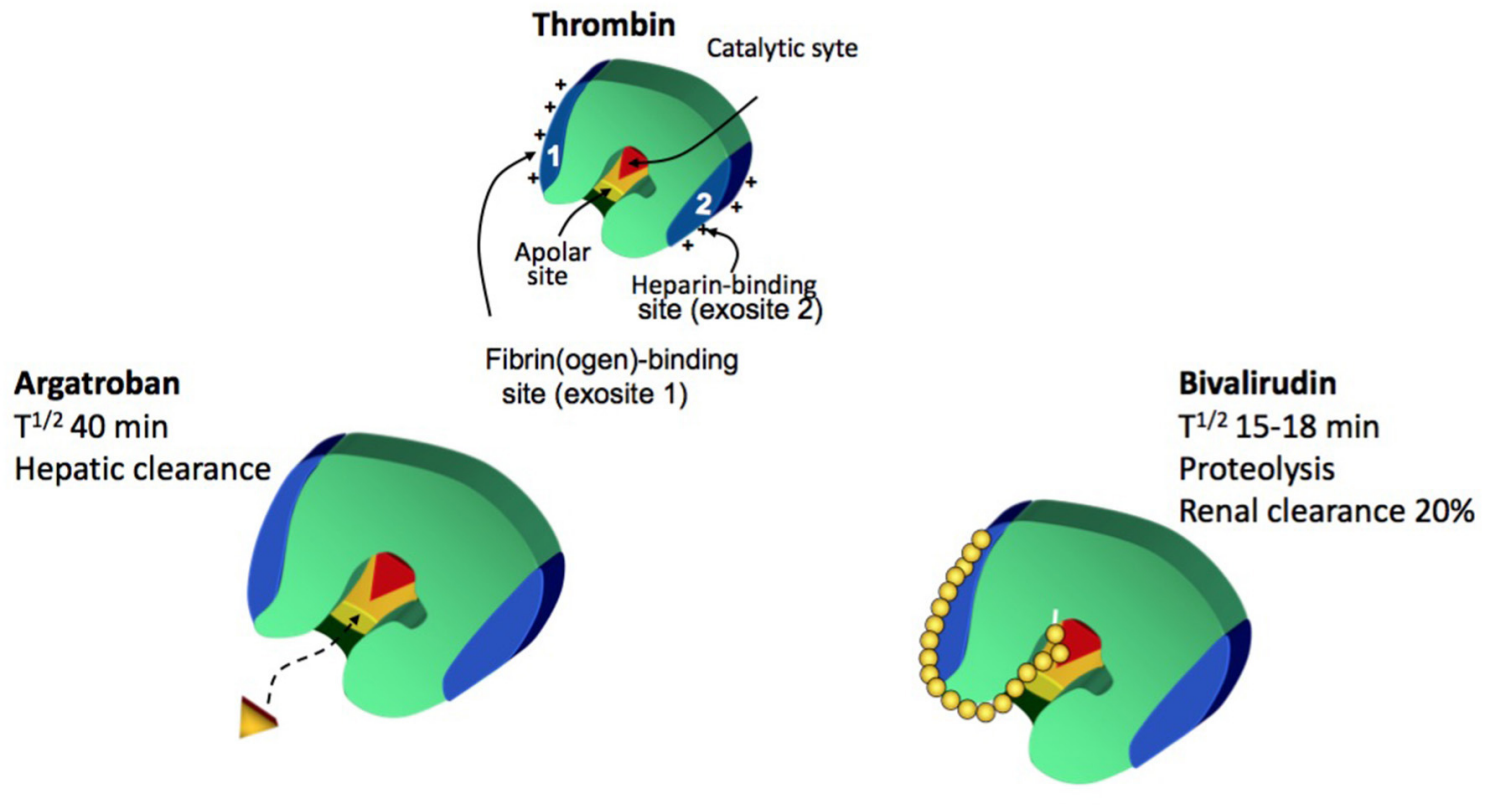
Direct Thrombin inhibitors mechanism (Modified by Patricia Massicotte. Warkentin TE. Best Practice & Research Clinical Haematology, 2004).

Thrombin-inhibiting drugs can block the action of thrombin by binding to its three domains: the catalytic and two exosites. Exosite 1 binds fibrin while exosite 2 binds the AT–heparin complex (52). Bivalent DTIs, such as bivalirudin, block thrombin at both the catalytic site and exosite 1, whereas univalent DTIs, such as argatroban, bind only to the catalytic site. The fibrin–heparin–thrombin complex results in a steric hindrance and prevents further binding of heparin–AT molecules, making the AT–heparin complex a poor inhibitor for clot-bound thrombin (52) (Figure 3). Differently, DTIs can also inhibit thrombin bound to fibrin, given their AT-independent action. By reducing the thrombin-mediated activation of platelets, DTIs may also have an antiplatelet effect. Provided that DTIs do not bind to plasma proteins, these anticoagulants may have more predictable effect than UFH and LMWH (52).

### Pharmacokinetics and Pharmacodynamics

The safety of DTIs, which are predominantly cleared by the kidneys, remains to be established in patients with renal dysfunction. The half-life of bivalirudin is prolonged in subjects with severe renal impairment, and decreased doses are needed. Hepatic metabolism and proteolysis at other sites also contribute to bivalirudin metabolism. The use of aspirin does not affect the plasma concentrations of DTIs (52).

Further studies on bivalirudin pharmacodynamics and pharmacokinetics in pediatrics would be necessary.

Table 4 provides a list of the route of administration, plasma half-lives, and main sites of clearance of the various DTIs.

**Table 4:** Pharmacokinetic characteristics of DTIs.

**Characteristic**	**Bivalirudin**	**Argatroban**	**Dabigatran**
Route of administration	Intravenous	Intravenous	Oral
Plasma half-life	25 min	45 min	12 h
Main site of clearance	Kidney, liver, other sites	Liver	Kidney

### Bivalirudin Use in Pediatric ECMO and VAD

The use of bivalirudin in pediatric ECMO has been poorly evaluated, and currently, no prospective studies on bivalirudin use in MCS exist. This is due to the fact that most of the studies include heparin as first-line therapy. In literature, different indications for bivalirudin have been reported, but the most frequent reasons are heparin resistance or diagnosis of HIT.

Ranucci et al. reported that bivalirudin use in postcardiotomy ECMO was characterized by significantly higher ACT, aPTT, and R values at TEG, compared to heparin. The authors described patterns of fluctuation in heparin responsiveness requiring dose adjustments accordingly to low AT and correction-related modification of AT levels. The AT-independent bivalirudin antithrombotic action would be responsible for stably higher values of anticoagulation tests. Moreover, patients treated with bivalirudin received lower amount of allogeneic transfusions. The initial bivalirudin infusion rate reported was 0.03–0.05 mg/kg/h, with a medium maintenance rate of 0.05–0.1 mg/kg/h (53).

Nagle et al. analyzed the bivalirudin use in pediatric VV and VA ECMO. In order to maintain a therapeutic aPTT, initial bivalirudin infusion doses ranged from 0.05 to 0.3 mg/kg/h, while maintenance infusion rate needed ranged from 0.045 to 0.48 mg/kg/h. This strategy was associated to an average percentage of time on therapeutic aPTT range of 47.5% and no cerebrovascular bleeding events occurred (54).

Ezetendu et al. described a case report of shift from heparin to bivalirudin to manage anticoagulation because of VV ECMO-related hemolysis and hyperbilirubinemia precluding accurate monitoring of heparin therapy (aPTT >150 s with anti-Xa level was < 0.1 IU/ml). Bivalirudin was successfully used for anticoagulation in this infant, with an initial dose of 0.3 mg/kg/h. No bolus was needed (55).

Preston et al. reported replacement of heparin with bivalirudin in a pediatric patient requiring VV-ECMO who developed HIT. The bivalirudin loading doses were between 0.4 and 1.6 mg/kg and the maintenance dose ranged from 1.2 to 1.8 mg/kg/h. The authors described the efficacy of continuous bivalirudin infusion in case of plasma exchange during VV ECMO (56).

Pollak et al. described the use of bivalirudin in a newborn, which showed HIT-related thrombosis after 3 days of VA-ECMO support for congenital diaphragmatic hernia. Bivalirudin was administered at a loading dose of 0.4 mg/kg. Then, the initial infusion rate of 0.15 mg/kg/h was titrated to maintain the ACT in the target range of 180–200 s, requiring a dose ranging between 0.06 and 0.17 mg/kg/h. Of note, while the initial efficacious infusion rate was very low, an escalation dose to 1.1–1.6 mg/kg/h was necessary to maintain a target level (57).

Campbell et al. retrospectively reviewed 34 pediatric patients receiving bivalirudin for ECMO/VAD. The average dose of bivalirudin infusion was 0.37 mg/kg/h, with a maximum dose of 0.62 mg/kg/h. This is the first study to report that even though bivalirudin dosing in ECMO and VAD patients is consistent with a previously described dosing, bivalirudin requirement may be higher in VAD patients (58).

In a recent case series of continuous-flow left VADs, Sylvia et al. demonstrated that bivalirudin seems to be associated with a lower risk of major bleeding (59).

Hamzah et al. first reported a different starting dose of bivalirudin according to renal clearance and described a strategy to briefly reach a therapeutic goal in anticoagulation level. To reach the aPTT goal target, an initial dose of bivalirudin was 0.3 mg/kg/h in case of creatinine clearance > 60 ml/min, instead of 0.15 mg/kg/h for subjects with renal dysfunction. Infusion rate was titrated by 0.05–0.1 mg/kg/h dose increment. Bleeding events were fewer in the bivalirudin group and there was no difference in the rate of thrombotic events between the two groups. Furthermore, the authors demonstrated how the requirement of bivalirudin needed to maintain target anticoagulation increased over time according to increasing creatinine clearance and plasmatic fibrinogen levels. The authors concluded that the use of bivalirudin in pediatric MCS is feasible, safe, reliable, and cost-effective in comparison to heparin (60).

VanderPluym et al. first described DTI use in pediatric paracorporeal VAD support in North America, reporting a lower incidence for major bleeding and stroke events than that previously evidenced. Forty-three pediatric patients, aged < 19 years, received bivalirudin or argatroban. The type of device implanted included Berlin Heart EXCOR (49%), paracorporeal continuous-flow devices (44%), and combination of devices (7%). Initial infusion rate ranged between 0.1 and 0.4 mg/kg/h, with an average of 0.3 mg/kg/h. The maximum dose ranged between 0.1 and 3.9 mg/kg/h with a median value of 1 mg/kg/h. The target aPTT value was variable between 50 and 100 s. Major bleeding was reported in 16% of patients and stroke in 12% of cases (1.7 events per 1,000 patient days of support on DTI). The overall survival to transplantation was 88% (61).

Finally, Bates et al. highlighted the potential use of bivalirudin, in a heterogeneous VAD population, in case of suspected or confirmed pump thrombosis. The authors reported the safety of the drug (no correlation between bivalirudin and death was described) and a good management of this fearsome adverse event with bivalirudin. Only one case of major bleeding occurred and the patient made a full recovery (62).

In general, the results of all the above mentioned studies are encouraging and promising. Nevertheless, further specific well-designed, randomized, prospective control trials between the standard of care with UFH and DTIs in pediatric MCS setting are needed.

Finally, some form of DTI resistance has been suspected, probably related to the possibility to develop neutralizing antibodies as well as peptides. However, at the moment, we do not exactly know the mechanism by which sensitivity to DTIs could be reduced or even lost over time (63).

### Protocols of Antithrombotic Strategies

Recently, conventional antithrombotic regimens in patients requiring VAD or ECMO are progressively changing, contextually to the changes in antithrombotic international protocols.

Regarding the common antithrombotic strategies for MCS, the Edmonton Anticoagulation and Platelet Inhibition Protocol was the first detailed guideline that provides the mainstay antithrombotic regimen in pediatric paracorporeal VADs for a long time (49). It was based on conventional antithrombotic drugs such as UFH, LMWH, and warfarin and antiplatelet therapy with aspirin and clopidogrel. The heparin dose was adjusted according to anti-Xa levels of 0.35–0.5 units/ml and corresponding aPTT levels 1.5–2.5 times the subject's baseline. Antiplatelet therapy was initiated according to TEG platelet mapping (Haemonetics, Brain-tree, MA) parameters (49).

The investigational Device Exemption (IDE) evaluated the Edmonton protocol outcomes and related events. This analysis, published by Steiner et al., showed an incidence of 24% for major bleeding events and 9% for neurologic complications. Most patients suffering from major events were inside the therapeutic range proposed by the Edmonton protocol (64). Major bleeding events were associated to an ASA inhibition and anticoagulation levels above the target range only in 25 and 22% of cases, respectively. Neurologic events were correlated to excessive antithrombotic therapy intensity in 9% of times. Thus, the authors suggested for a more aggressive strategy with earlier initiation of ASA and a more rapid increase of dose titration for the use of agents with alternative mechanism of action and different methods of monitoring antiplatelet effects (64). Indeed, according to the suggestions of the IDE trial, the original Edmonton Protocol has been progressively changed.

Regarding modifications of antiplatelet therapy, Rosenthal et al. evaluated an anti-thrombotic regimen based on greater platelet inhibition with less reliance on platelet function testing.

In this study (64), children supported with the EXCOR at Stanford from 2009 to 2014 were divided into two cohorts. Children implanted before 2012 on TEG platelet mapping-modulated double anti-platelet strategy (Edmonton cohort) were compared to those implanted after September 2012 on routinely used triple anti-platelet therapy (Stanford modified anti-thrombotic guideline cohort). In the Stanford modified cohort, antiplatelet drug doses were adjusted to high, weight-based dosing targets, and in case of signs of inflammation, low-dose steroids were administered.

The targeted doses of aspirin, clopidogrel, and dipyridamole were higher and less variable in the Stanford cohort (65). The incidence rate of stroke in the Stanford cohort was 84% lower than in the Edmonton one (0.8 vs. 4.9 events per 1,000 days of support) and 86% lower than in the previous IDE trial. Also, the bleeding risk was lower in the Stanford group.

Recently, the use of DTIs has become increasingly common in antithrombotic strategies revealing encouraging findings. The first and largest multi-center experience of DTI use in pediatric Excor VAD support, described by VanderPluym et al. and previously cited, was based on an antithrombotic strategy using bivalirudin or argatroban associated with adjuvant antiplatelet therapy. A lower rate of stroke compared to the Edmonton guideline was reported with risk for adverse events approximating those reported for intracorporeal continuous-flow VADs in children with older age (61, 66). Furthermore, a higher incidence rate of stroke (1.2 vs. 0.8 events per 1,000 days of support) and a lower incidence of bleeding (2.6 vs. 8.6 events per 1,000 days of support), compared to the strategy adopted by Rosenthal et al. (based on enoxaparin with triple anti-platelet regimen), were reported.

Taking into consideration the various logistical, ethical, and financial challenges of completing a randomized controlled trial of anticoagulation agents in the pediatric VAD population, MCS centers worldwide are seeking learning collaborations.

In April 2017, the Advanced Cardiac Therapies Improving Outcomes Network (ACTION) was created (67). This network was focused on generating a registry data on goals and outcomes of pediatric assist devices in order to facilitate connectivity and to share data between different centers. Since ACTION was created, standardized protocols for bivalirudin administration and monitoring have been designed and adopted by the vast majority of pediatric MCS institutions. As reported by Peng, over 30 centers reached an agreement to change their practice and followed the updated protocols produced by ACTION. Part of this design is even to educate the patients and their family to make children dischargeable at home.

North America data on approaches to issues, such as pre-implant evaluations and adverse events occurring after implantation, were “harmonized,” offering a protocol for pulsatile paracorporeal VADs (BERLIN HEART EXCOR^®^).

This protocol helps team decisions at the bedside. The goals of aPTT values are suggested according to the risk level for thrombosis and bleeding. Ranges of initial dosing of bivalirudin and its titration to maintain a therapeutic range of aPTT, such as strategy of transition from DTIs to vitamin K antagonist (VKA), are described.

In case of high bleeding risk, as in the early period post-implantation, the aPTT goal of 50–60 s is recommended. For standard risk of thrombosis, bivalirudin titration should be adjusted to maintain aPTT between 60 and 80 s; for high risk of thrombosis, an aPTT goal between 70 and 90 s is suggested (68). Any adjustment of the dose is recommended by a simple titration rule (Table 5).

**Table 5:** ACTION Harmonization recommendations for bivalirudin infusion for VADs.

**Initial bival dosing**	**maintenance bival titration**
**Goal: aPTT** ***High risk (of bleeding)**: aPTT 50–60 s*	**Goal: aPTT** • ***Standard risk:** aPTT 60–80 s* • ***High risk (of thrombosis):** aPTT 70–90 s*
Renal function (GFR)	***If aPTT 5 to 15 s out of range:*** • Increase or decrease by 15% (round up to closest 2nd decimal) • Recheck 2–3 h after dose change
Normal (>60 ml/min/1.73 m^2^) 0.3 mg/kg/h IV infusion	***If aPTT in target range, no change***. • Recheck after 2–3 h, then can decrease frequency when stable
Mild-moderate (30–60 ml/min/1.73 m^2^) 0.2 mg/kg/h IV infusion	***If aPTT ≥15–30 s out of range*** • Increase or decrease by 25% (round up to closest 2nd decimal) • Recheck 2–3 h after dose change
Severe (< 30 ml/min/1.73 m^2^) 0.1 mg/kg/h IV infusion	***If aPTT >3 × baseline or ~120 s:*** • With normal renal function: hold 15 min and reduce by 30% • With mild to moderate renal dysfunction: hold for 45 min and reduce by 40% • With severe renal dysfunction: hold 2 h and recheck PTT before restarting

*aPTT, activated partial thromboplastin time; bival, bivalirudin; GFR, glomerular filtration rate*.

Moreover, this protocol provides recommendations for adjuvant antiplatelet therapy, indicating doses and adverse effects, how to titrate the therapy, how to modify it on the basis of clinical signs (fibrin/thrombin deposition in the circuit and bleeding), and responsiveness testing.

## Conclusions

The topic of MCS remains a hot issue in the pediatric setting and is the center of many debates. There are many reasons behind this complexity: first, the difficulty to manage the adverse events of pediatric patients supported with ECMO or assisted with temporary or durable devices. Second, even though there are advancements in biocompatibility, the prevention or treatment of complications, such as bleeding and thrombosis, remains challenging.

Nowadays, the efforts of collaboration between various centers with the aim to harmonize different practices have the potential to result in changes in clinical management. Collaborative ACTION could have a positive impact on the outcome and could bring a significant contribution in supporting clinicians on anticoagulation policies. Without undermining the need for prospective randomized controlled trials able to compare anticoagulation agents in pediatric MCS, it is fundamental to highlight the importance of current observational reports. Undoubtedly, there are immense difficulties to complete these studies, in terms of logistical, ethical, and financial challenges.

Based on the literature presented above, this review concludes that, aligning with the protocols of ACTION, the use of bivalirudin seems safe with a simple learning curve. In case it is handled according to anticoagulation therapeutic range, it facilitates the uniformity of treatment protocols in different centers. To sum up, the use of bivalirudin appears to be effective to control thromboembolic complications even in younger infants who underwent different types of MCS.

## Author Contributions

CG, AR, and LDC wrote 100% of the manuscript. IF, AA, FC, SGP, ML, and GDF revised the manuscript. All authors contributed to the article and approved the submitted version.

## Conflict of Interest

The authors declare that the research was conducted in the absence of any commercial or financial relationships that could be construed as a potential conflict of interest.

## Publisher's Note

All claims expressed in this article are solely those of the authors and do not necessarily represent those of their affiliated organizations, or those of the publisher, the editors and the reviewers. Any product that may be evaluated in this article, or claim that may be made by its manufacturer, is not guaranteed or endorsed by the publisher.
